# Ultrasonic-Assisted Synthesis and Cytocompatibility Assessment of TiO_2_/SiO_2_ Nanoparticles-Impregnated Gum Arabic Nanocomposite: Edible Coating of Dates for Shelf-Life Extension

**DOI:** 10.3390/polym17020161

**Published:** 2025-01-10

**Authors:** Jegan Athinarayanan, Vaiyapuri Subbarayan Periasamy, Ali A. Alshatwi

**Affiliations:** Nanobiotechnology and Molecular Biology Research Laboratory, Department of Food Science and Nutrition, College of Food Science and Agriculture, King Saud University, Riyadh P.O. Box 2460, Saudi Arabia; jegan@ksu.edu.sa (J.A.); psubbarayan@ksu.edu.sa (V.S.P.)

**Keywords:** dates, nanostructures, edible coating, cytocompatibility, biosilica

## Abstract

The post-harvest management of fruit is crucial to preventing its decay and loss. Generally, edible coatings are applied to fruit to avoid decay and microbial contamination. We have used ultrasonication to synthesize TiO_2_ and *Pennisetum glaucum* residue-derived biosilica embedded in gum arabic nanocomposite. The SiO_2_/TiO_2_/gum arabic nanocomposite morphological and crystalline features were investigated using a scanning electron microscope and X-ray diffractometer, respectively. The SiO_2_/TiO_2_/gum arabic cytocompatibility was assessed using cell viability and microscopic assay. The SEM images revealed that 70–90 nm biosilica and 70–100 nm TiO_2_ nanostructures were present on the gum arabic. According to MTT assay and microscopic examination results, SiO_2_/TiO_2_/gum arabic do not inhibit cell viability and modulate cellular structural features; it inferred that SiO_2_/TiO_2_/gum arabic possess good cytocompatibility on human mesenchymal stem cells even up to 400 µg/mL. The date fruits were immersed in SiO_2_/TiO_2_/gum arabic-based coating mixtures and stored at 6 °C for 4 weeks. When date fruits were examined during storage, it was found that the applied coatings contributed to maintaining physicochemical features (e.g., color and texture). These findings suggest that the SiO_2_/TiO_2_/gum arabic-based coating can be applied to extend the shelf life of dates.

## 1. Introduction

Postharvest processing removes undesirable elements, enhances fruit appearance, and ensures quality [1,2]. A postharvest technique involves monitoring and controlling temperature, humidity, packaging, and fungicides [3,4]. Postharvest management aims to improve the value of fruits and vegetables [5]. The value of fruits and vegetables is added continuously until they can be eaten after harvesting [6]. Increasing wages and keeping fruit fresh throughout the year are benefits of postharvest technologies [7,8]. Earlier studies have shown that 30–40% of fruits and vegetables are thrown away because of damage or spoilage [9,10]. Several technological advancements can reduce postharvest losses. Edible coatings are an effective and novel method for preserving fruits and vegetables to reduce postharvest loss [11]. Using edible coatings is expected to extend the shelf life and maintain the quality of meat, fish, dairy, fruits, and vegetables. In recent years, edible films and coatings have emerged as a promising technology to enhance food safety by preventing environmental exposure and increasing shelf life [11,12]. Fruit coatings derived from natural ingredients also reduce spoilage rates and preserve freshness during transportation and storage [13]. The edible coatings can also serve as delivery systems of functional ingredients like antibacterials, odors, antioxidants, and nutritional components, extending the freshness of fruits and preventing microbial growth [14]. In addition, edible coatings are commonly applied to different fruits, including bananas, papayas, tomatoes, grapes, mangoes, strawberries, avocados, apples, oranges, and seasonal fruits [13,15,16,17].

Dates are seasonal fruits that grow widely in semi-arid and dry climates. In Arab countries, it is widely recognized as a crucial tree. Producing date fruits provides significant economic assistance to native communities [18]. Date fruit is enriched with various nutrients, including fiber, carbohydrates (fructose and glucose), minerals, and vitamins [19,20,21]. There are many challenges and obstacles to maintaining the quality and extending the shelf life of dates, such as environmental and biological factors [22]. It has been reported that 5–20% of dates were lost during postharvest management [23]. In date fruit post-harvesting management, microbial contamination is the primary concern. Dates deteriorate quickly due to their moisture, oxygen, and chemical composition [24]. In recent years, studies have been conducted on developing edible coatings for preserving and maintaining the freshness of date fruit. For instance, Rahemi et al. reported that olive oil, methylcellulose, and pectin-based edible coatings were utilized to protect the semi-dry and dry dates [25]. Abu-Shama et al. reported that guar gum, gelatin, chitosan, and composite-based edible coatings prolong Barhi date fruit freshness [18]. Additionally, soy protein, gelatin, zein, chitosan, thyme oil, aniseed oil, pea starch, and carnauba wax were used in the edible coating of dates [26,27,28].

Organic/inorganic nanohybrid materials have garnered attention for their electrical, catalytic, antimicrobial, antioxidant, anticancer, and cytocompatible properties. Le et al. reported that ZnO/chitosan/gum arabic edible coating showed promising results for the shelf-life extension of avocados up to 7 days at room temperature [29]. According to Ramírez-Concepción et al. study, the chitosan/TiO_2_/ZnO/MgO composite coating most effectively prolonged the shelf life of jackfruit bulbs [30]. Gum arabic is a natural polymer that contains a non-viscous soluble fiber derived from exudates of acacia species [31]. It is extensively employed as a stabilizer and emulsifier in pharmaceutical, food, and cosmetic applications [32]. It is interesting to note that gum arabic possesses antibacterial, antioxidant, antidiabetic, and antiulcer activities. In traditional medicine, gum arabic is also used to relieve inflammation of the intestinal mucosa and kidney disease [31,32,33]. Nanoparticles of TiO_2_ and SiO_2_ exhibit unique physical, chemical, and biological properties, which improve the composite material’s performance. Specifically, TiO_2_ nanoparticles have photocatalytic, antibacterial, and UV-shielding properties [34]. The SiO_2_ nanoparticles were used as an anti-caking agent, gas and moisture barrier, reinforcing filler [35]. Some studies have reported that nanomaterials incorporated into polymers can improve their tensile strength and elasticity [35,36]. The role of organic/inorganic nanohybrids-based edible coating materials on date fruits at the khalal stage is unknown. Thus, in this study, we fabricated the SiO_2_/TiO_2_/gum arabic nanocomposite via ultrasonication and employed them as an edible coating formulation. Furthermore, we assessed the preservation effectiveness of SiO_2_/TiO_2_/gum arabic nanocomposite edible coatings on dates at the khalal stage.

## 2. Materials and Methods

### 2.1. Raw Materials and Chemicals

The *Pennisetum glaucum* residues were purchased from Karur, Tamil Nadu, India. Glycerol, TiO_2_ nanoparticles, gum arabic, and hydrochloric acid were purchased from Sigma Aldrich, St. Louis, MO, USA. We collected dates at the khalal stage from a local date palm farm in Riyadh, Saudi Arabia. Fetal bovine serum (FBS), Dulbecco’s Modified Eagle Medium (DMEM), 3-(4,5-dimethylthiazol-2-yl)-2,5-diphenyltetrazolium bromide dye, ethidium bromide (EB), acridine orange (AO), and JC-1 stain were obtained from Thermo Fisher Scientific, Waltham, MA, USA.

### 2.2. Synthesis of Biogenic Silica Nanostructures

We mixed 10 g of *Pennisetum glaucum* residues powder with 100 mL of 0.1 M hydrochloric acid [37]. Afterward, we transferred the mixture to an autoclave and kept it at 120 °C under 103 kPa of pressure for 2 h. Subsequently, acid-pretreated seed hull residues were extracted by filtration. After that, distilled water was used to wash the residues until the acid was removed. Then, the residue was dried and calcined in a muffle furnace at 500 °C for 1 h.

### 2.3. Synthesis of SiO_2_/TiO_2_ Embedded in Gum Arabic

Silica nanostructures (60 mg) derived from *Pennisetum glaucum* residues and TiO_2_ nanoparticles (40 mg) were mixed with 200 mL of 5% gum arabic aqueous solution. Next, the mixture was sonicated at 750 W at 20 kHz for 30 min using a probe ultrasonicator (VCX-750, Sonics, Newtown, CT, USA). The obtained colloid was dried at 60 °C. Following that, the obtained material was used for further studies.

### 2.4. Characterization of SiO_2_/TiO_2_ Embedded in Gum Arabic

We investigated the functional groups of gum arabic and their SiO_2_/TiO_2_ hybrid using FTIR measurements (Bruker Alpha, Eco Corporations, Berlin, Germany). Using a thermogravimetric analyzer, we analyzed the decomposition and weight loss profiles of gum arabic and their SiO_2_/TiO_2_ hybrid as the temperature increased (TGA-60H Shimadzu, Kyoto, Japan). Thermogravimetric analysis was performed at a temperature range of 30–600 °C at a rate of 10 °C/min. We investigated the differences in the crystallinity profiles of gum arabic and their SiO_2_/TiO_2_ hybrid using an X-ray diffractometer operating with CuK α-radiation (Lab X, XRD 6100, Shimadzu, Kyoto, Japan) and 2θ data acquired ranging from 10 to 80°, scan speed: 5° min^−1^.

### 2.5. Cytocompatibility Assessment

The impact of SiO_2_/TiO_2_-embedded gum arabic powder on human mesenchymal stem cell viability was examined using the MTT assay described previously [38]. The 96-well plate was plated with around 1 × 10^4^ cells per well. Following 80% confluence, the cells were exposed to different doses of testing material for 24 and 48 h. After exposure, 20 µL of MTT dye at 5 mg/mL concentration in phosphate buffer saline (PBS) was poured into each well of the plate, and the plate was incubated for 4 h. After carefully aspirating the cell culture media, 200 µL of dimethyl sulfoxide (DMSO) was added to each well. The absorbance of the plate was measured using a microplate reader at 570 nm and 650 nm. Cell viability of human mesenchymal stem cells was calculated from the obtained results.

### 2.6. Microscopic Assessment of Cell Morphology

We examined the effects of SiO_2_/TiO_2_-embedded gum arabic on human mesenchymal stem cells using bright-field and fluorescent microscopes [39]. The 24-well plate was seeded with approximately 1 × 10^5^ cells per well. After reaching 80% confluence, the cells were incubated with various SiO_2_/TiO_2_-embedded gum arabic concentrations for 24 and 48 h. A bright-field microscope was used to examine the cellular morphology after incubation, and photographs were taken. Fluorescent microscopic analysis of the cells was performed by staining them with acridine orange/ethidium bromide (AO/EB), examining them under a microscope, and capturing pictures.

### 2.7. Coating

We collected dates during their khalal stage. We discarded dates that were physically damaged or decayed. We chose dates with uniform color and size. After that, dates were equally divided into two groups. The dates were washed and dried at an ambient temperature. The SiO_2_/TiO_2_-impregnated gum arabic coating formulation was prepared using 1% glycerol through ultrasonication. Following complete mixing, the coating formulation was used for date coating. We soaked the dates in coating formulation for 2 min, then dried them at an ambient temperature for 30 min. Afterward, the coated dates were stored in a polyethylene terephthalate box with venting holes at 6 °C. All analyses used uncoated dates as controls under the same storage conditions.

### 2.8. Color

We monitored the color changes of date fruit after edible coating with a colorimeter (CM-5, Minolta, Tokyo, Japan). The color of dates fruit was estimated using three color parameters: L* (lightness), a* (green/red), and b* (blue/yellow). Date fruit color features were examined from three different points, using their means to evaluate the color variation (E), yellowness index (YI), whiteness index (WI), and chroma.

### 2.9. Firmness

We assessed the firmness of date fruits at different intervals during storage using the Brookfield CT3 texture analyzer (Brookfield, Middleboro, MA, USA). A two-cycle test measured the textural profile (hardness, springiness, and cohesiveness).

### 2.10. Statistical Analysis

Statistical analysis was performed from three replicates’ data for each measurement. The statistical analysis was performed using Microsoft Office Excel 2016. The statistical significance between the control and coated group was investigated using the student-t test. Also, the significance was considered as *p* ≤ 0.05.

## 3. Results and Discussion

**Figure 1:**
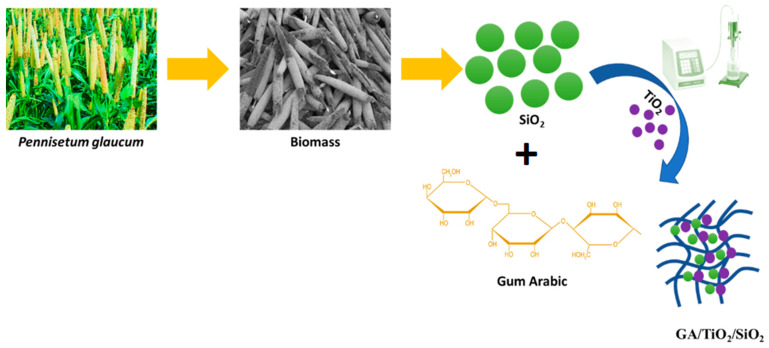
Schematic representation of SiO_2_/TiO_2_/gum arabic fabrication.

Hybrid organic–inorganic systems comprising organic and inorganic materials gained considerable attention in food packaging systems because of their promising physicochemical features. Several hybrid organic/inorganic materials exist in nature, including bone, mollusk shells, and teeth, formed from biomacromolecules and inorganic ingredients assembled at a nanoscale [40]. As a result of the synergistic interactions between organic and inorganic functions, a nanocomposite possesses new properties, including enhanced electrical, catalytic, optical, magnetic, and antimicrobial attributes. In this study, we synthesized biogenic silica and TiO_2_ nanoparticles-impregnated gum arabic nanocomposite using an environmentally benign approach. Figure 1 depicts the schematic diagram of gum arabic with biogenic SiO_2_/TiO_2_ hybrid fabrication.

Silica phytoliths are deposited in plant tissues through the silicification mechanism. A significant silica content is found in plant residues. Accordingly, some studies have reported the fabrication of silica nanostructures from agrarian waste [41,42]. In this study, silica nanostructures were fabricated from pearl millet seed husk. Utilizing inexpensive, non-toxic, and natural *Pennisetum glaucum* (pearl millet), seed husk is an excellent raw material for naturally occurring silica. Also, seed husks from pearl millet consist of 9.1% silica [37]. Pearl millet biomass-derived silica nanostructures and TiO_2_ nanoparticles were mixed with gum arabic and sonicated at 750 W for 30 min. Ultrasonication is a highly versatile dispersing strategy employed in several applications. The process involves breaking down large particles into smaller or more uniformly sized ones [43]. Under sonication, the SiO_2_ and TiO_2_ nanostructures dispersed well and adhered to the gum arabic matrix (Figure 1).

**Figure 2:**
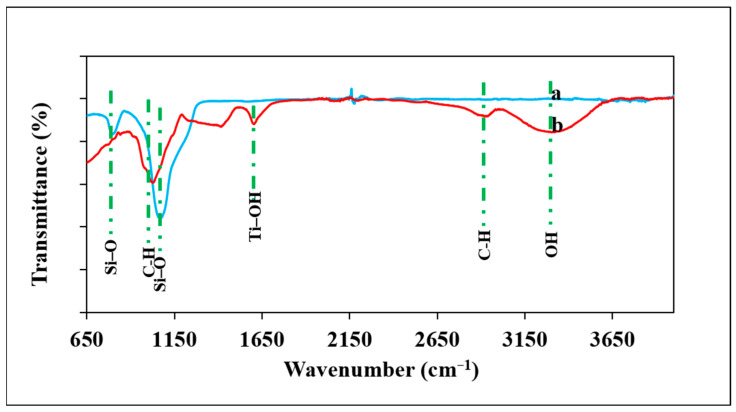
FTIR spectra of (**a**) *Pennisetum glaucum*-derived biogenic silica and (**b**) SiO_2_/TiO_2_/gum arabic nanocomposite.

The FT-IR spectra of the SiO_2_ nanoparticles and TiO_2_/SiO_2_ incorporated gum arabic are shown in Figure 2. The biogenic silica nanoparticles show notable peaks at 1167, 1070, and 810 cm^−1^, vibration of Si–O–Si, asymmetric vibration of Si–O, and symmetric vibration of Si–O, respectively (Figure 2a). The spectrum of TiO_2_ nanoparticles showed peaks around 3448, 1639, 686, and 532 cm^−1^ (Supporting Material Figure S1). The 3448 and 1639 cm^−1^ peaks correspond to the OH group of water and deformative vibration of the Ti–OH stretching mode of TiO_2_ nanoparticles. The spectrum of SiO_2_/TiO_2_/gum arabic showed the band at 3309 cm^−1^ ascribed to extensive intramolecular hydrogen bonds and stretching vibration of the OH groups (Figure 2b). The peak at 2931 cm^−1^ is attributable to the vibration mode of the C–H bonds. The 1604 cm^−1^ peaks correspond to the deformative vibration of the Ti–OH stretching mode of TiO_2_ nanoparticles. Two characteristic bands at 1418 cm^−1^ and 1244 cm^−1^ correspond to C=O symmetric and C–O–C stretching vibration. A distinct peak at approximately 1067 cm^−1^ corresponds to the alkene C–H bending in gum arabic. Additionally, we observed that the peaks around 1141, 988, and 810 cm^−1^ are responsible for Si–O–Si, asymmetric Si–O vibration, and symmetric Si–O, respectively (Figure 2b). These results revealed the presence of TiO_2_ and SiO_2_ nanoparticles on gum arabic matrix.

**Figure 3:**
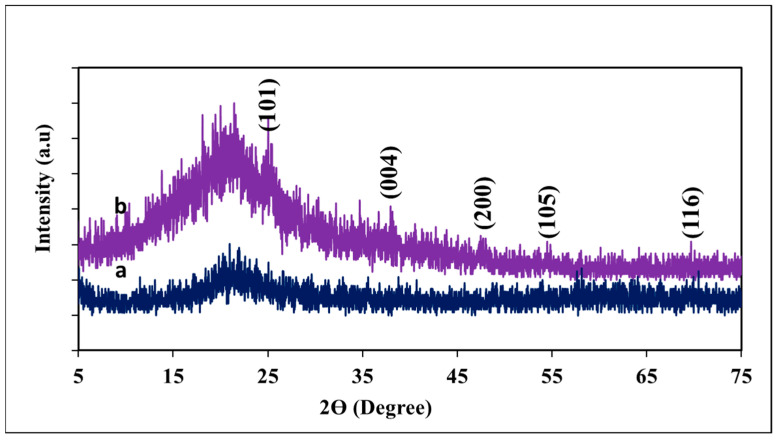
XRD pattern of (**a**) *Pennisetum glaucum*-derived biogenic silica and (**b**) SiO_2_/TiO_2_/gum arabic nanocomposite.

X-ray diffraction (XRD) patterns of silica nanoparticles and SiO_2_/TiO_2_/gum arabic nanocomposite are present in Figure 3. The silica nanoparticles exhibited the peak at 2θ = 21.12°, revealing the presence of amorphous silica nanoparticles (Figure 3a). This observation is consistent with previous researchers who have noted the amorphous nature of silica [37]. Upon incorporating SiO_2_ and TiO_2_ nanostructures, a broad peak was observed between 19 and 24.92°, corresponding to gum arabic and silica. Also, we examined different additional peaks at 2θ of 25.02°, 37.94°, 47.98°, 54.82°, and 69.68°, which are responsible for anatase TiO_2_ lattice planes of (1 0 1), (0 0 4), (2 0 0), (1 0 5), and (1 1 6), respectively. Our results suggest that highly crystalline TiO_2_ and amorphous silica nanoparticles were successfully impregnated onto the gum arabic matrix.

**Figure 4:**
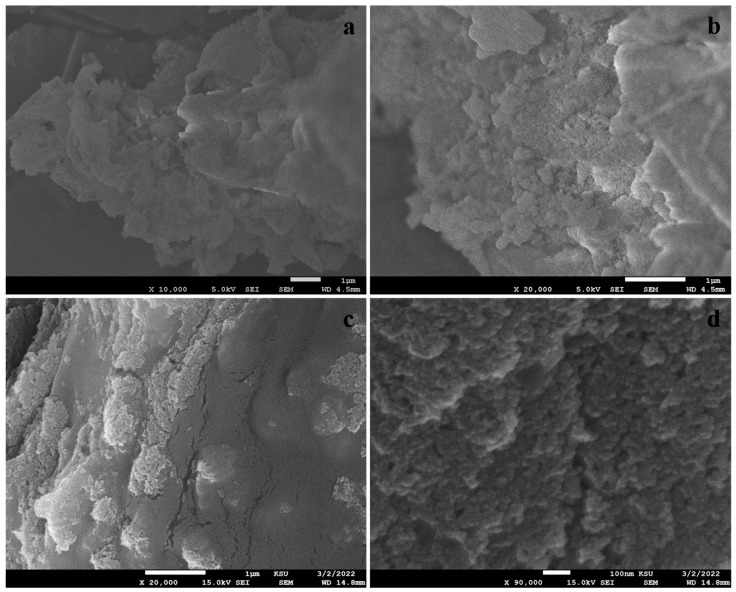
SEM images of (**a**,**b**) *Pennisetum glaucum*-derived biogenic silica and (**c**,**d**) SiO_2_/TiO_2_/gum arabic nanocomposite.

**Figure 5:**
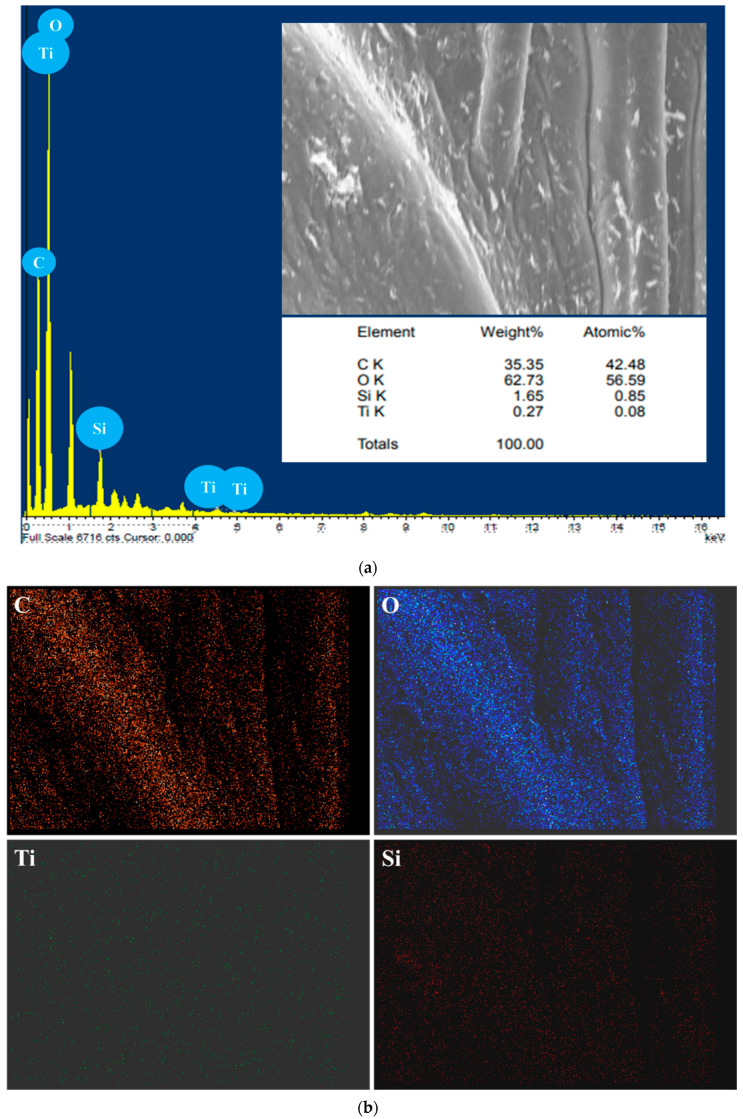
(**a**) Energy-dispersive X-ray diffraction (EDX) spectrum of SiO_2_/TiO_2_/gum arabic nanocomposite and (**b**) elemental mapping of prepared SiO_2_/TiO_2_/gum arabic nanocomposite.

Figure 4 presents SEM images of biogenic silica nanoparticles and TiO_2_ and SiO_2_ incorporated gum arabic matrix. The SEM images display spherical silica nanoparticles of 70–90 nm in size in Figure 4a,b. Moreover, the SiO_2_/TiO_2_/gum arabic SEM images possess 70–100 nm spherical TiO_2_ and SiO_2_ nanoparticles distributed evenly on the gum arabic. These results are attributed to the strong interaction between TiO_2_/SiO_2_ and gum arabic (Figure 4c,d). Figure 5a displays the elemental composition of the prepared SiO_2_/TiO_2_/gum arabic nanocomposite. The results confirmed the presence of Ti, Si, O, and C elements in SiO_2_/TiO_2_/gum arabic nanocomposite. Also, the elemental mapping image indicates that Si and Ti elements dispersed well on the gum arabic matrix (Figure 5b). Compared with organic materials, the amount of inorganic ingredients (SiO_2_ and TiO_2_) is significantly less (Figure 5a) in the prepared nanocomposite. Thermogravimetric analysis results of gum arabic and SiO_2_/TiO_2_/gum arabic nanocomposite are shown in Figure S2. We observed the weight loss percentage of gum arabic reached about 81%. The first stage of weight loss for gum arabic occurs between 100 and 260 °C, as the water molecules are dehydrated. The second weight loss occurred between 260 °C and 350 °C, which is associated with the degradation of hydrocarbons. The weight loss in SiO_2_/TiO_2_/gum arabic was around 71%. Based on the results, the SiO_2_/TiO_2_-impregnated gum arabic showed superior thermostability.

**Figure 6:**
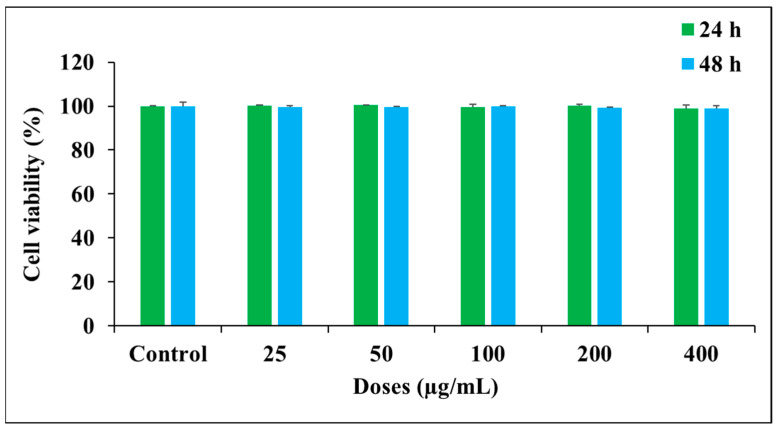
Influence of prepared nanocomposite on cell viability of human mesenchymal stem cells assessed using MTT assay.

The biological compatibility of materials is essential for its application in the food industry. Thus, we studied SiO_2_/TiO_2_/gum arabic hybrid materials’ biological compatibility with human mesenchymal stem cells. The SiO_2_/TiO_2_/gum arabic cytocompatibility was evaluated using various techniques, including cell viability, cellular and nuclear morphology, and JC-1 staining. We assessed the viability of hMSCs after SiO_2_/TiO_2_/gum arabic exposure. These results revealed that SiO_2_/TiO_2_ nanoparticles-incorporated gum arabic did not affect cell viability. Compared to the control, SiO_2_/TiO_2_/gum arabic nanocomposite did not affect cell viability after 24 h and 48 h exposure (Figure 6). These findings imply that hMSCs survived successfully with SiO_2_/TiO_2_/gum arabic nanocomposite. Cell and material interaction can be improved by the hydrophilicity of gum arabic in nanocomposite. Earlier studies demonstrated that SiO_2_ nanoparticles have good biocompatibility [37].

**Figure 7:**
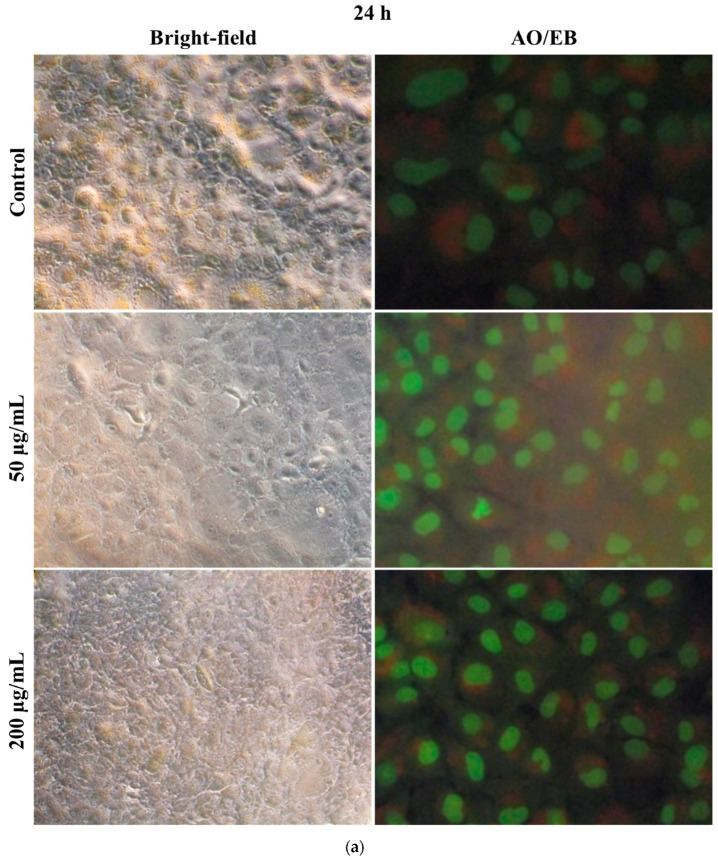

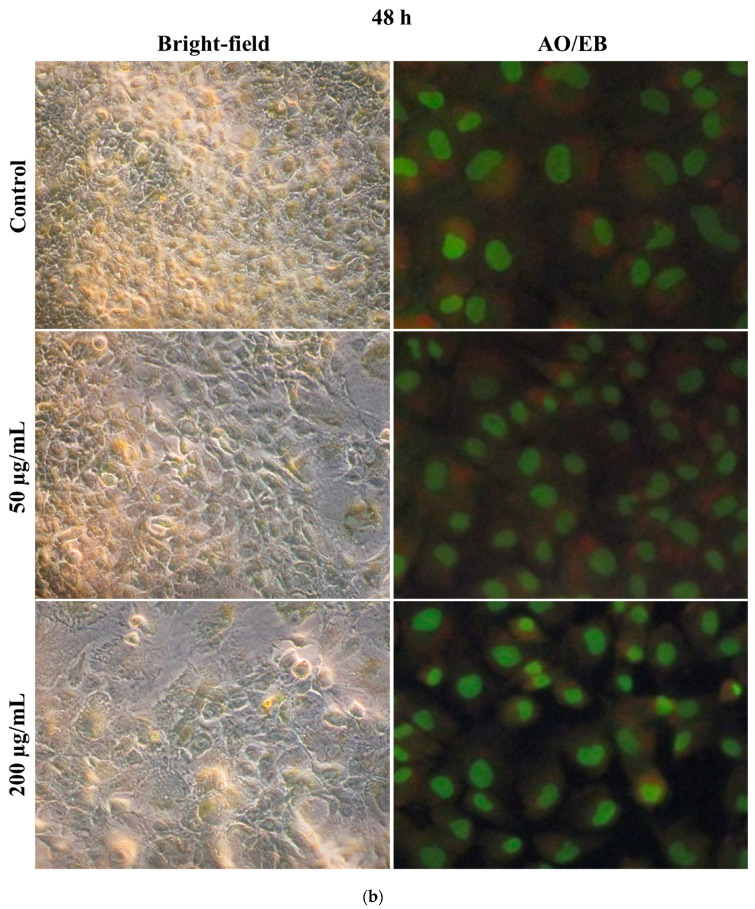
(**a**) Bright-field microscopic and acridine orange/ethidium bromide staining images of human mesenchymal stem cells after 24 h exposure to SiO_2_/TiO_2_-incorporated gum arabic nanocomposite. (**b**) Bright-field microscopic and acridine orange/ethidium bromide staining images of human mesenchymal stem cells after 48 h exposure to SiO_2_/TiO_2_-incorporated gum arabic nanocomposite.

Microscopic observation of cell structural changes in hMSCs after 24 and 48 h SiO_2_/TiO_2_/gum arabic exposure was studied. Figure 7 displays bright-field and fluorescence microscopic images of hMSCs after SiO_2_/TiO_2_/gum arabic exposure. The cell morphology examination revealed the cells were healthy and grew well in SiO_2_/TiO_2_/gum arabic-treated and a control group of 24 and 48 h. Also, acridine orange/ethidium bromide (AO/EB) staining illustrated the cells were viable (Figure 7a,b). We did not find any dead cells in the treated and control groups. Our result suggested that SiO_2_/TiO_2_/gum arabic nanocomposite can promote hMSCs proliferation and good growth.

**Figure 8:**
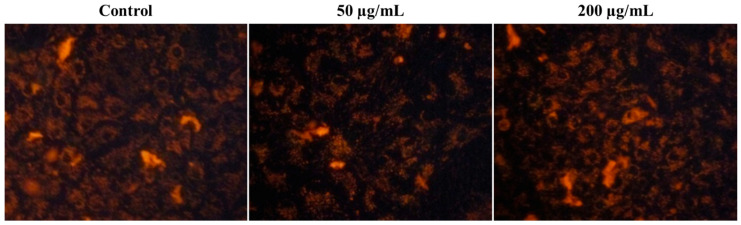
Effect of SiO_2_/TiO_2_-incorporated gum arabic nanocomposite on the mitochondria membrane potential of human mesenchymal stem cells.

Furthermore, we investigate the mitochondrial membrane potential (MMP) level in SiO_2_/TiO_2_/gum arabic-treated and control hMSCs to find cell health. Figure 8 represents MMP changes in SiO_2_/TiO_2_/gum arabic-treated and control groups. These study results indicated no significant difference between the control and treated groups. The SiO_2_/TiO_2_/gum arabic nanocomposite is also non-toxic. Thus, SiO_2_/TiO_2_/gum arabic nanocomposite can be used in food industrial applications.

Due to the biocompatibility of SiO_2_/TiO_2_-incorporated gum arabic nanocomposite, we utilize it for date fruit shelf-life extension and preservation via an edible coating approach. Generally, food color is one of the main critical components in attracting consumers. Fruit and vegetable colors are also a reliable sign of ripeness, freshness, quality, and maturity. Most commonly, edible coatings do not interfere with fruit and vegetable colors. In particular, in this study, we have examined the outer color of raw and SiO_2_/TiO_2_/gum arabic nanocomposite-coated khalal stage dates at various storage stages at 6 °C. Table 1 illustrates the color change results of khalal stage dates. Color differences between the control and treated groups after a week of storage were insignificant. Notably, the control group showed a change in color after 2 weeks. After 4 weeks, we found a significant color change in the control group compared with SiO_2_/TiO_2_/gum arabic nanocomposite-coated dates. All the color parameters have noticeable changes between the treatment and control groups because it is easy to make predictions based on individual color parameters (i.e., L*, a*, and b*).

**Figure 9:**
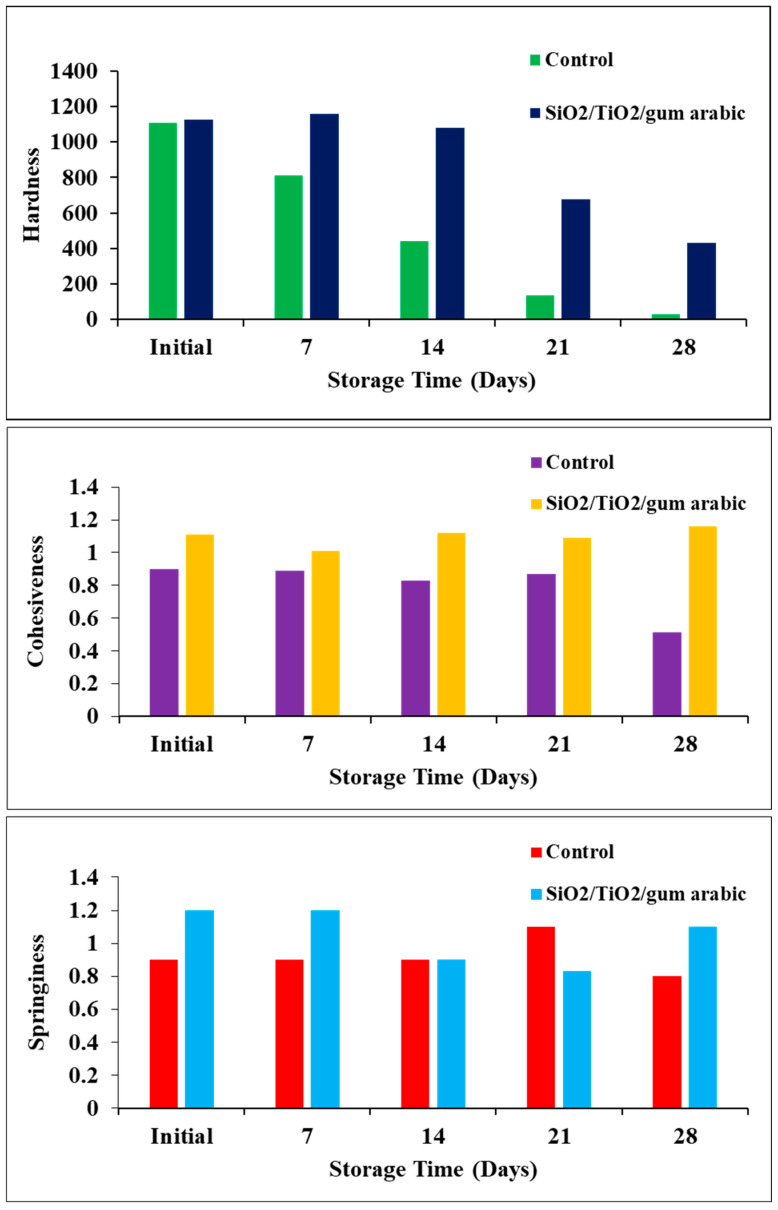
Textural features of SiO_2_/TiO_2_/gum arabic nanocomposite-coated and uncoated khalal stage dates during storage at 6 °C.

**Figure 10:**
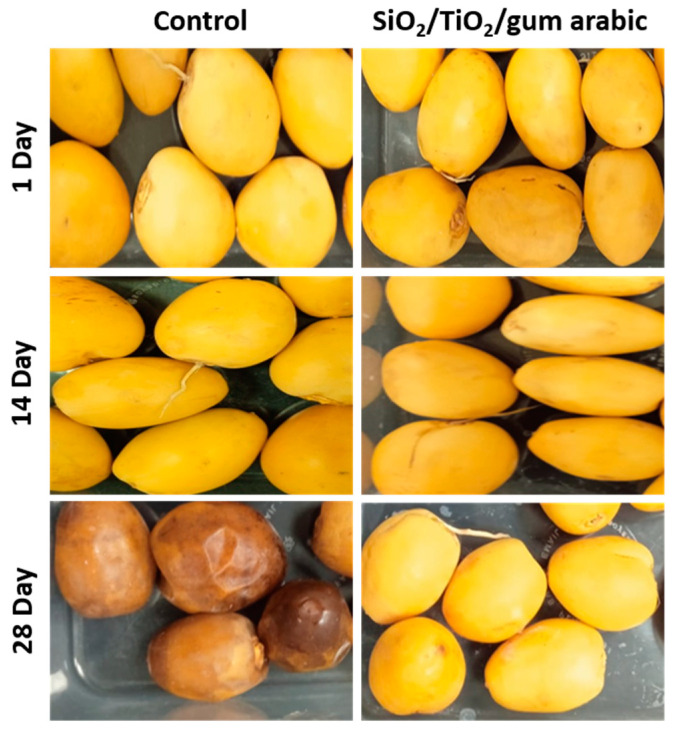
Digital images of SiO_2_/TiO_2_/gum arabic nanocomposite-coated and uncoated khalal stage dates during storage at 6 °C.

A fruit’s texture is also a crucial aspect that contributes to its freshness and quality. Several factors can affect fruit texture, including moisture content, chemical composition, and the ripening stage. According to the texture study, the control sample’s hardness decreased significantly over time in coated and uncoated dates (Figure 9). Meanwhile, the SiO_2_/TiO_2_/gum arabic-coated dates have higher hardness than the control dates. Additionally, cohesiveness and springiness were evaluated in this study. Coated dates had slightly improved cohesiveness and springiness. Earlier studies reported that different biopolymers, including guar gum, gelatin, and chitosan-based edible coatings, extend Barhi date fruit freshness [18]. Furthermore, soy protein, zein, thyme oil, aniseed oil, pea starch, and carnauba wax were used as edible coatings [26,27,28]. Based on our findings, we found that SiO_2_/TiO_2_/gum arabic nanocomposite coatings extended the shelf life of dates. The rate of senescence and ripening process may increase during storage, which leads to a loss of hardness (Figure 10). Hence, based on this significance, future studies will measure the chemical composition and enzyme activity using different coating formulations containing SiO_2_/TiO_2_/gum arabic.

## 4. Conclusions

We have synthesized TiO_2_/SiO_2_ nanoparticles embedded in gum arabic nanocomposite via ultrasonication. The SEM images revealed biosilica nanostructures of 70–90 nm and TiO_2_ nanostructures of 70–100 nm adhered on gum arabic. Microscopic analysis and MTT assay results indicated that SiO_2_/TiO_2_/gum arabic maintained cell viability and did not modulate cellular structural features. Therefore, it is concluded that SiO_2_/TiO_2_/gum arabic possessed good cytocompatibility on human mesenchymal stem cells even at 400 g/mL. We coated date fruits with SiO_2_/TiO_2_/gum arabic nanocomposite and stored them at 6 °C. During storage, it was found that the applied coatings contributed to maintaining the physicochemical features of dates. As a result of these findings, it seems that a coating based on SiO_2_/TiO_2_/gum arabic can be used to prolong the shelf life of dates.

## Figures and Tables

**Table 1:** Color parameters in SiO_2_/TiO_2_ nanostructures-impregnated gum arabic-coated khalal stage dates at different storage intervals.

Storage Time (Days)	L*		a*		b*	
	Control	SiO_2_/TiO_2_/gum Arabic	Control	SiO_2_/TiO_2_/gum Arabic	Control	SiO_2_/TiO_2_/gum Arabic
0	56.97	62.63	8.22	7.49	34.64	40.08
7	54.75	62.05	9.26	5.13	35.54	36.98
14	53.34	61.42	7.36	6.93	37.71	42.62
21	43.70	59.78	7.11	8.15	22.67	42.71
28	34.45	54.07	5.85	8.23	15.92	38.41

## Data Availability

The original contributions presented in this study are included in the article/Supplementary Material. Further inquiries can be directed to the corresponding author.

## Supplementary Materials

The following supporting information can be downloaded at https://www.mdpi.com/article/10.3390/polym17020161/s1, Figure S1: FTIR spectrum of TiO_2_ nanoparticles; Figure S2: Thermogravimetric analysis of (a) gum arabic and (b) SiO_2_/TiO_2_/gum arabic nanocomposite.
