# Supplementary material for: Ultrasonic-Assisted Synthesis and Cytocompatibility Assessment of TiO2/SiO2 Nanoparticles-Impregnated Gum Arabic Nanocomposite: Edible Coating of Dates for Shelf-Life Extension
Source: Polymers (Basel). 2025 Jan 10;17(2):161. doi: 10.3390/polym17020161 (PMC11769035; doi:10.3390/polym17020161)
Supplement: Supplementary file 1 [file polymers-17-00161-s001.zip › polymers-3370321-supplementary.pdf]

Ultrasonic-Assisted Synthesis and Cytocompatibility Assessment of TiO_2/SiO_2 Nanoparticles Impregnated Gum Arabic Nanocomposite: Edible Coating of Dates for Shelf-life Extension

Jegan Athinarayanan, Vaiyapuri Subbarayan Periasamy and Ali A Alshatwi *

Nanobiotechnology and Molecular Biology Research Laboratory, Department of Food Science and Nutrition, College of Food Science and Agriculture, King Saud University, P.O. Box 2460, Riyadh, Saudi Arabia
*Correspondence: Tel: +966 1 467 7122; Fax: +966 1 467 8394. E-mail: nano.alshatwi@gmail.com; alshatwi@ksu.edu.sa)

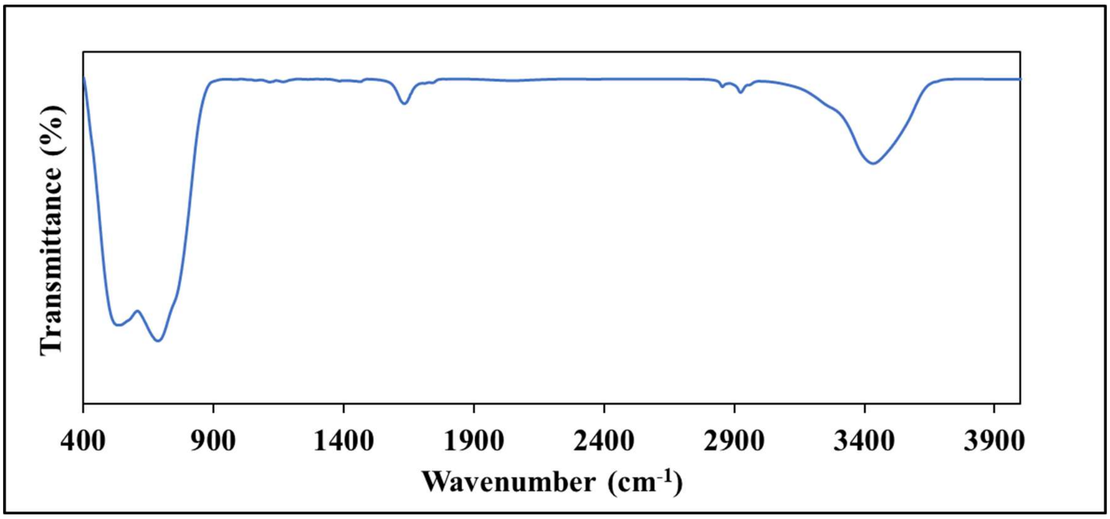

Figure S1: FTIR spectrum of TiO_2 nanoparticles

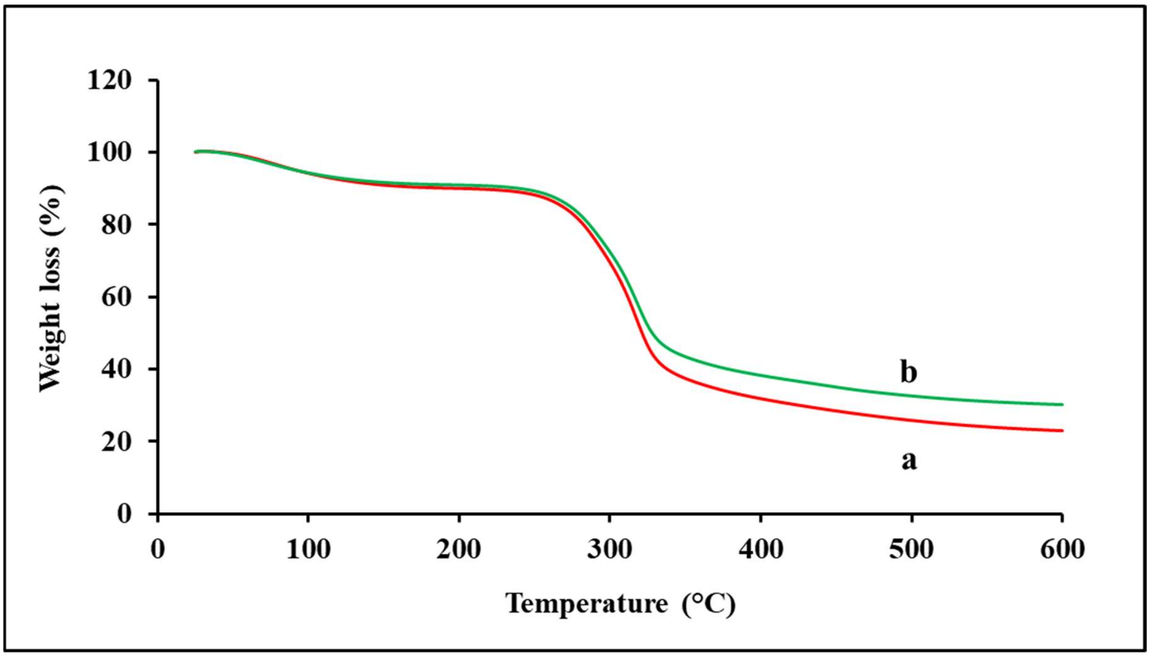

Figure S2: Thermogravimetric analysis of (a) gum arabic and (b) SiO_2/TiO_2/gum arabic nanocomposite
